# Validity of a new assessment rubric for a short-answer test of clinical reasoning

**DOI:** 10.1186/s12909-016-0714-1

**Published:** 2016-07-26

**Authors:** Euson Yeung, Kulamakan Kulasagarem, Nicole Woods, Adam Dubrowski, Brian Hodges, Heather Carnahan

**Affiliations:** 1 Department of Rehabilitation Sciences, University of Toronto, 160-500 University Avenue, Toronto, ON M5G 1V7 Canada; 2 Department of Family and Community Medicine, University of Toronto, Toronto, Canada; 3 Department of Surgery, University of Toronto, Toronto, Canada; 4 Division of Emergency Medicine, Memorial University of Newfoundland, St John’s, Canada; 5 Faculty of Medicine, University of Toronto, Toronto, Canada; 6 School of Human Kinetics and Recreation, Memorial University of Newfoundland, St John’s, Canada; 7 The Wilson Centre for Research in Education, University Health Network, Toronto, Canada; 8 Wilson Centre for Research in Education Richard and Elizabeth Currie Chair in Health Professions Education Research, University Health Network, Toronto, Canada

**Keywords:** Certification, Assessment, Clinical reasoning, Short-answer test, Validity, Physical therapy, Orthopaedic

## Abstract

**Background:**

The validity of high-stakes decisions derived from assessment results is of primary concern to candidates and certifying institutions in the health professions. In the field of orthopaedic manual physical therapy (OMPT), there is a dearth of documented validity evidence to support the certification process particularly for short-answer tests. To address this need, we examined the internal structure of the Case History Assessment Tool (CHAT); this is a new assessment rubric developed to appraise written responses to a short-answer test of clinical reasoning in post-graduate OMPT certification in Canada.

**Methods:**

Fourteen physical therapy students (novices) and 16 physical therapists (PT) with minimal and substantial OMPT training respectively completed a mock examination. Four pairs of examiners (*n* = 8) participated in appraising written responses using the CHAT. We conducted separate generalizability studies (G studies) for all participants and also by level of OMPT training. Internal consistency was calculated for test questions with more than 2 assessment items. Decision studies were also conducted to determine optimal application of the CHAT for OMPT certification.

**Results:**

The overall reliability of CHAT scores was found to be moderate; however, reliability estimates for the novice group suggest that the scale was incapable of accommodating for scores of novices. Internal consistency estimates indicate item redundancies for several test questions which will require further investigation.

**Conclusion:**

Future validity studies should consider discriminating the clinical reasoning competence of OMPT trainees strictly at the post-graduate level. Although rater variance was low, the large variance attributed to error sources not incorporated in our G studies warrant further investigations into other threats to validity. Future examination of examiner stringency is also warranted.

## Background

The primary aim of certification examinations in the health professions is to restrict clinical practice to those who demonstrate adequate competence within a particular clinical domain [1]. Consequences of candidates’ success or failure for certification examinations have enormous implications for the candidate, institutions granting the certification and the patients that we serve. For example, mistakenly passing candidates with inadequate competence could potentially pose significant risks to patients’ health and safety. Certifying institutions must therefore make every effort to ensure that the decisions derived from assessment results are well supported by sound, scientific evidence of validity [2].

Validity refers to the extent to which the conclusions drawn from the assessment instrument in question are justifiable, relevant and meaningful for a specific purpose [3]. Accordingly, determining the validity of assessment results in the certification context requires a chain of inferences that collectively signals the extent to which interpretations of examination results are trustworthy [3].

In order to generate a coherent series of inferences that can ultimately support the dependability of high-stakes decisions required for certification, validity evidence must be examined and gathered in a systematic manner [4]. Theories of validity provide a framework with which to formulate and test such inferences about the interpretation of assessment results. Evidence of validity can therefore be generated from five distinct sources: instrument content; response process; internal structure of the instrument; relationships between assessment scores and other variables; and the consequences of decisions made based on the assessment results (Table 1) [5].

**Table 1:** Sources of validity evidence (adapted from Messick 1995, Andreatta and Gruppen 2009 and Cook and Beckman 2006)

Source of validity evidence	Description of validity evidence	Method of generating validity evidence for the CHAT
1.Instrument content	Extent to which instrument content is relevant to the construct of interest	Developing a test blueprint for clinical reasoning in OMPT
2.Response process	Extent to which the cognitive and physical processes required by the instrument can represent the construct of interest	Examining feasibility and acceptability of scoring procedure
3.Instrument’s internal structure	Extent to which the transformation of assessment results into a score reflects the underlying construct	Calculating internal consistency Establishing inter-rater reliability Conducting factor analysis
4.Relationships between assessment scores and other variables	Extent to which assessment results relate with other variables that possess a predicted association with the construct of interest	Examining correlation with other clinical reasoning measures
5.Consequences of decisions made based on assessment results	Evidence pertaining to intended and unintended consequences of interpreting and using assessment results	Establishing and examining method of determining pass/fail on case history examination

### Generating validity evidence for OMPT certification

To generate the requisite validity evidence for a particular interpretation of assessment results the context for which the interpretations are intended must be well defined; otherwise the validity evidence may be inappropriate and thus irrelevant [6]. In the field of orthopaedic manual physical therapy (OMPT), a post-graduate subspecialty within physical therapy, there is a dearth of published validity evidence to support the assessment results derived for certification purposes. Given that OMPT includes the practice of relatively high-risk procedures such as spinal manipulations for which sub-standard practices may result in harm to patients [7], current OMPT certification procedures stand to be improved and substantiated by high quality validity evidence.

Competence in clinical reasoning is explicitly assessed as a portion of the certification process worldwide [8, 9]. In Canada, clinical reasoning is assessed, in part, through a written short-answer examination that is based on a standardized clinical scenario. At present, the manner in which candidates’ written responses to this test is evaluated by assessors and the interpretation of the associated scores lack sufficient validity evidence; thus, the dependability of test results may be vulnerable to scrutiny by stakeholders. Moreover, research conducted on rater-based assessments has largely focused on rater performance on assessments such as the Objective Structured Clinical Examination (OSCE), with little attention paid to other rating tasks including the assessment of short-answer written tests.

To address this important gap in the literature, we undertook the development of the Case History Assessment Tool (CHAT); this is an assessment rubric designed to evaluate candidates’ clinical reasoning competence through the appraisal of written responses to the short-answer test administered for OMPT certification in Canada [10]. The CHAT was developed to improve the manner in which the construct of clinical reasoning is assessed through a short-answer test in OMPT. The CHAT was based on a previously published clinical reasoning assessment framework that describes the construct of clinical reasoning in OMPT; details regarding the development of this framework are published elsewhere [11].

To date, validity evidence has been generated regarding the content and response process associated with using the CHAT [12]. The purpose of the present study was to generate further validity evidence related to the instrument’s internal structure in order to strengthen the chain of inferences that demonstrate the trustworthiness of interpretations of examination results (Table 1). Specifically, we aimed to answer the following questions about the scores generated from the CHAT:

1. To what extent can scores be generalized from participants with novice level clinical reasoning ability to participants with higher level of clinical reasoning ability in OMPT?
2. To what extent can scores be generalized from one rater to another (inter-rater reliability)?

## Methods

### Assessment rubric

The OMPT written short-answer test in Canada is based on a standardized patient scenario, and aims to assess candidates’ clinical reasoning competence. This test contains a total of 16 open-ended test questions that assess OMPT-specific knowledge and clinical reasoning skills including hypothesis testing, interpretation of patient data, design of a management plan, and justification for candidates’ decisions. While the questions for this short-answer test remain the same from year to year, a different patient scenario of similar level of difficulty is used each year. Two examiners independently appraise each candidate’s test paper.

To improve current methods, the CHAT was developed to assess the written responses to this test in order to determine candidates’ clinical reasoning competence in a more standardized and comprehensive manner [11]. The assessment items within the CHAT were constructed and assigned to each of the 16 short-answer questions based on the clinical reasoning domain(s) represented in each question. For example, written responses pertaining to the primary hypotheses are evaluated using multiple assessment items concerned with how well the hypotheses account for all of the patient data, and the accuracy and comprehensiveness of the candidates’ justification. Thus, multiple and varying numbers of assessment items are used to evaluate written responses to each of the 16 short-answer questions depending on the aspect of clinical reasoning being evaluated.

In total, the CHAT contains 45 assessment items that utilize a 5-point Likert scale with narrative descriptors assigned to ratings 1, 3 and 5. The narrative descriptors were specifically worded to reflect the domain(s) of clinical reasoning being represented by the short-answer question. A rating of 3 on the 5-point Likert scale indicates a passing grade for all items in the assessment rubric. A composite score is then calculated based on previously established weighting for each of the 16 test questions; weighting for each question was determined by a national group of physiotherapist examiners who achieved consensus through a rigorous process that was underpinned by international education standards in OMPT. In addition, 4 global rating statements with a similar 5-point Likert scale were developed to capture a more holistic appraisal of candidates’ clinical reasoning.

### Study design

Two participant groups were invited to complete a mock written examination that approximated the content and usual procedures of the Canadian OMPT written short-answer test: physiotherapists who were preparing for the OMPT certification examination (‘PT’ group) and final year physiotherapy students who have completed all orthopaedic courses in the entry-to-practice program at the University of Toronto (‘novice’ group). We use the term ‘experience’ to describe the two participant groups; study participants with substantial OMPT training, and therefore assumed to have greater clinical reasoning ability (‘PT group’), and those with minimal OMPT training (‘novice group’), and hence assumed to have lower levels of clinical reasoning ability. Physiotherapist participants were recruited through various examination preparatory sessions or courses. Due to issues of convenience, physiotherapist participants completed the written test through self-invigilation within a 3-h period convenient to them. Completed examinations were submitted electronically and anonymized prior to assessment by two examiners independently. Physiotherapy students completed the same mock examination over a 3-h period through in-person invigilation, after which test papers were similarly anonymized and forwarded to 2 examiners for independent assessment. It was hypothesized that systematic differences in clinical reasoning competence exist between these two groups and that the CHAT is capable of detecting these differences.

Examiners registered with the Canadian Physiotherapy Association were recruited to complete a 20-min online training module prior to rating the test papers. Test papers were divided amongst 4 pairs of participating examiners. Due to resource limitations and reasons of feasibility, each pair of examiners was given a different set of written test papers for independent rating. Examiners were instructed to rate each test paper using the CHAT. Numeric scores were recorded in an Excel spreadsheet and forwarded to study investigators.

### Data analysis

Generalizability theory (G theory) was used as a framework for evaluating the dependability of the scores generated from the CHAT. Specifically, we used G theory to characterize how accurately test scores from the CHAT permit generalization to the candidate’s clinical reasoning competence under the measurement conditions in the Canadian OMPT certification context. G theory offers many advantages in this context. Firstly, while it makes assumptions of normality, these assumptions are ‘looser’ and have been shown to be robust given that it makes estimates using analysis of variance procedures. Thus, it can be applied to most data with confidence that the results will indeed be reflective of larger samples and repeated assessments. Secondly, the focus of this study was on the characteristics of the *test,* i.e., *the reproducibility of the scores.* In other words, we wished to understand the facets contributing error to scoring and whether optimization of reliability was possible. More broadly, our study focused on aggregated performance of raters, cases, items in terms of error. In these circumstances generalizability theory provides robust estimates and addresses the central research questions [13]. The benefits of Multi-faceted rasch modelling (MFRM) have also been argued for examining such assessment data. MFRM is a useful technique and focuses on the reproducibility of ability estimates by modelling the contribution of multiple facets (e.g., raters, items) whereas the traditional or single facet rasch analysis examines the contribution of only items. Although MFRM is useful for detailing measurement error, it has stricter assumptions of the data that cannot always be met. In this case, individual items are independent of cases or raters which is problematic for model fitting. Given the focus of this study, MFRM was considered less appropriate than G theory.

Data analysis oriented around G theory helped the authors gain a deeper understanding of the deployment of measurement resources and measurement points in order to maximize the reliability of scoring the written short-answer test in OMPT certification. We believe that G theory is more efficient and intuitive and additionally afforded us direct comparison between sources of error variance with other similar assessments.

In the present study, the facet of differentiation was the examination candidate, or *person* (p), which was nested in level of education *experience* (e) (Table 2). Other facets of generalization were defined as *rater*, *question*, and *item* (nested within *question*). Conceptually, each test score in a generalizability analysis is exchangeable with all possible observations taken from that measurement scenario. Under such assumptions, facets of generalization are typically treated as random effects. However, in the case of the CHAT, *question* and *items* remain fixed from year to year.

**Table 2:** Defining facets in a G study

Facet	Description of facet	In the present study	
		Name of facet	Number of levels of the facet
Facet of differentiation	The source of variation associated with the object of measurement	Candidates	30 candidates
Facet of generalization	The sources of variation associated with all other relevant factors in the measurement scenario	Raters	8 raters (4 pairs of raters)
		Experience	2 levels of education experience (Novice and PT groups)
		Questions	16 short-answer test questions
		Items	45 assessment items

First, we used the observed test scores to conduct a generalizability study (G study) with all participants analyzed as one group, and with participants nested into the stratification of *experience*. This generated variance estimates that approximated the magnitude of each of the identified source of variance relevant to our research questions. As the purpose of the CHAT is to differentiate between candidates with high and low levels of clinical reasoning ability, a relatively large variance due to *person* was desired. The resulting variance estimates were then used to calculate reliability coefficients and standard errors, which estimated the overall generalizability of scores and provided a sense of measurement precision respectively. Separate G studies were subsequently conducted with test scores from the novice and PT groups in order to determine separate reliability estimates for these two groups. We additionally conducted an analysis of variance of aggregate scores for these two groups to estimate the effect size of differences in the observed scores.

To respect the structure of the data collection, separate G studies were additionally conducted for each rater pair. We used classical test theory to approximate the confidence interval around the generalizability coefficients as there is no agreed upon method for this calculation [14].

Due to the size of the variance estimates for the *question* effect, we also estimated the internal consistency reliability for test questions with two or more assessment items. Cronbach’s alpha was calculated for assessment items associated with these questions in order to estimate internal consistency.

Finally, we used the data from the G study to conduct *decision* studies (D study) in order to design the optimal application of the CHAT for OMPT certification. This was accomplished by increasing or decreasing the levels of one or more of the facets of generalization and estimating the associated reliability for hypothetical measurement scenarios [15].

## Results

In total, 16 physiotherapists and 14 physiotherapy students completed the written short-answer test. Eight examiners, with a mean of 13.88 years (SD = 3.92) of examination experience, participated as raters. All participants provided informed written consent to participate in this study.

Table 3 reports the variance components and generalizability coefficient (0.749) that indicate moderate reliability of the assessment results derived from the CHAT in our omnibus analysis. Candidates’ level of education *experience* accounted for 12.93 % of the total variance, reflecting systematic differences in clinical reasoning competence between novice and PT candidates in this sample as measured by the CHAT. All other variance components due to interactions involving the *experience* facet were relatively small, with the exception of the p*q:e (15.69 %) and p*r*q:e (13.34 %) variance. These larger variance components involving the facet *experience* represent the varying relative standing of candidates across questions and across raters within the novice and PT groups.

**Table 3:** Summary of effects, estimated variance components and reliability coefficients, and results of D-study (expected reliability for different measurement scenarios)

Effect	Variance component	df	MS	VC (with negative values set to ‘0’)	% variance
Experience/level of training (e)	σ^2^_(e)_	1	365.1019	0.22457	12.92556
p:e	σ^2^_(p:e)_	28	22.2046	0.1565	9.007661
rater (r)	σ^2^_(r)_	1	20.6960	0	0
Question (q)	σ^2^_(q)_	17	17.5271	0.0353	2.0312
Item within question (i:q)	σ^2^_(i:q)_	28	3.0545	0.0187	1.0746
experience*rater	σ^2^_(e*r)_	1	30.4104	0.0372	2.1382
experience*question	σ^2^_(e*q)_	17	8.9908	0.0795	4.5775
experience*item within question (ei:q)	σ^2^_(e*i:q)_	28	1.9765	0.0310	1.7837
person*rater:experience (pr:e)	σ^2^_(p*r:e)_	28	5.6480	0.0954	5.4932
person*question:experience (pq:e)	σ^2^_(p*q:e)_	476	2.5138	0.2725	15.6860
person*item:experience*question	σ^2^_(p*i:e*q)_	784	0.5783	0.0837	4.8198
rater*question	σ^2^_(r:q)_	17	2.7036	0.0286	1.6473
rater*item:question	σ^2^_(r*i:q)_	28	0.8331	0	0
experience*rater*question	σ^2^_(e*rq)_	17	0.6226	0	0
experience*rater*item:question	σ^2^_(e*r*i:q)_	28	0.8834	0.0316	1.8211
person*rater*question:experience	σ^2^_(p*r*q:e)_	476	0.9885	0.2319	13.3475
person*rater*item:experience*question	σ^2^_(p*r*i:e*q)_	784	0.4109	0.4108	23.6467
TOTAL variance				1.7374	100
G-coefficient (95 % confidence interval)	0.749				
Number of raters (random)	G-coefficient				
3 raters, 18 questions (fixed)	0.818				
4 raters, 18 questions (fixed)	0.857				
5 raters, 18 questions (fixed)	0.882				
6 raters, 18 questions (fixed)	0.900				
7 raters, 18 questions (fixed)	0.913				

The estimated variance component for *rater* was found to equal zero while the interactions involving this facet were marginal, indicating that raters’ performance was relatively stable across different questions and across the novice and PT groups.

The variance components for *question* and interactions involving this facet ranged from 2.0 to 5.5 %, reflecting that questions varied somewhat in difficulty level. The large p*r*i:e*q variance (23.64 %) represents the varying relative standing of candidates across raters and items within question, as well as other sources of error not incorporated in the G study.

As the variance for *question* and interactions involving *question* were non-negligible, Cronbach’s alpha was calculated for all test questions that were assessed using 2 or more assessment items to further analyze the internal consistency of these assessment items. Cronbach’s alpha for assessment items allocated to 4 of these test questions were found to be <0.70 (Table 5). Although the corrected item-total correlation values were acceptable (>0.30) [16], several of these correlations were found to be >0.70, suggesting that redundancies exist within the items assigned to assess these test questions. This is also reflected in the corresponding Cronbach’s alpha values when items were deleted; Cronbach’s alpha values were not significantly altered when each of the assessment items was in turn deleted from the analysis.

When separate analyses were conducted for the novice and PT groups, the generalizability coefficients equaled 0.203 (CI: 0.017, 0.376) and 0.657 (CI: 0.536, 0.752) respectively (Table 4). Moreover, a substantial difference was noted for the variance attributed to *person* between these groups (1.36 % in the novice group, 17.42 % in the PT group). An analysis of between-groups variance corroborates these results indicating that a statistically significant difference exists between the scores in the novice and PT groups (p < 0.001) with an effect size of 0.483 (p < 0.001). Although the variance components for *rater* were marginal in both groups, greater variance was attributed to *rater* in the PT group (3.60 %) compared to the novice group (0 %). Notably, the p*r*i:q variance observed in the novice group is twice that in the PT group; these relatively large values indicate that the varying relative standings of candidates across raters and items within question, as well as other sources of error not incorporated in the G study, contributed the greatest amount to the observed variance in both groups.

**Table 4:** Level of education experience: Summary of effects, estimated variance components and reliability coefficients

PT group (*n* = 16)				
Effect	df	MS (PT)	VC (PT)	% VC (PT)
p	15	35.1493	0.2761	17.4239
r	1	50.2609	0.0570	3.5970
q	17	12.3626	0.1075	6.7825
i:q	28	1.1671	0.0110	0.6924
pr	15	7.3319	0.1319	8.3238
pq	255	2.6669	0.3093	19.5244
pi:q	420	0.4812	0.0803	5.06634
rq	17	1.7434	0.0111	0.7025
ri:q	28	0.6554	0.0209	1.3204
prq	255	0.9652	0.2588	16.3313
pri:q	420	0.3206	0.3206	20.2357
			1.5844	100
G-coefficient (95 % confidence interval)	0.657 (0.536–0.752)			
Novice group (*n* = 14)				
Effect	df	MS (novice)	VC (novice)	% VC (novice)
p	13	7.2683	0.0186	1.3620
r	1	0.8455	0	0
q	17	14.1553	0.1236	9.0729
i:q	28	3.8639	0.0938	6.8854
pr	13	3.7050	0.0534	3.9186
pq	221	2.3371	0.2300	16.88143
pi:q	364	0.6905	0.08780	6.4392
rq	17	1.5828	0.0006	0.0448
ri:q	28	1.0611	0.0390	2.8626
prq	221	1.0155	0.2009	14.7450
pri:q	364	0.5150	0.5150	37.7886
			1.36274	100
G-coefficient (95 % confidence interval)	0.203 (0.017–0.376)			

Analyses conducted for data derived from each pair of raters yielded mixed coefficients that ranged from 0.59 to 0.76. These coefficients indicate moderate reliability of the assessment results generated from the CHAT when participant data were analyzed within each of the rater pairs [17]. Variance components generated from these analyses were similar to those from the omnibus analysis, with rater variance remaining as negligible and variance attributed to *question* ranging from 1.97 to 10.39 %.

Finally, the results from D-studies (Table 3) suggest that increasing the number of raters or questions would not result in higher reliability estimates for this sample.

## Discussion

The purpose of this study was to provide additional validity evidence in support of the scores generated from the CHAT. Specifically, we sought to generate validity evidence supporting the internal structure of the CHAT by examining the extent to which CHAT scores could be generalized from participants with novice and post-graduate levels of education experience in OMPT, and from one rater to another.

Our study results yielded moderate reliability [18] of assessment scores derived from the CHAT when all data were analyzed as one group. Our hypothesis that systematic differences in clinical reasoning competence exist between novice and more experienced participants was substantiated by the large variance components attributed to *experience* and the moderate effect size for the difference in *experience* between these two groups. When the same analyses were conducted separately for the novice and PT groups, the reliability estimate remained as moderate for the PT group (0.657), but was found to be poor for the novice group (0.203). This finding challenges our hypothesis that the scale within the CHAT possesses sufficient range to accommodate the distribution of scores in our sample. Low reliability in the novice group may be a consequence of two reliability threats [19]. First, the differences in reliability estimates may be explained by the different methods of test invigilation employed for the two groups. Moreover, it is possible that novices in our sample were at a level of ability that was too low to lead to meaningful CHAT scores. Although the approaches to OMPT training are similar between pre-licensure and post-graduate education contexts in Canada, clinical reasoning processes observed in novice physical therapists have been characterized primarily by hypothetico-deductive reasoning and differ from the diverse reasoning processes employed by physical therapists with greater expertise [20–22]. Thus, future validity studies may consider discriminating the clinical reasoning competence of OMPT trainees strictly at the post-graduate level. Finally, differences in reliability estimates may also reflect the differences in exam administration methods between the two groups. Specifically, greater motivation to succeed may have existed among participants in the physiotherapist group. Where possible, administration of the certification examination in future studies should remain as similar as possible in order to reduce motivation as an influencing factor.

Since there are often no definitive ‘correct’ answers for any given clinical reasoning task, the variance associated with the *rater* facet may reflect examiners’ individual interpretation and judgment of candidate responses. On the other hand, low variance components observed for the interaction terms involving the *rater* facet suggest strong reproducibility of test scores amongst examiners (inter-rater reliability), albeit examiner judgments may be consistent but incorrect. In contrast to generalizability studies of rater-based assessments that consistently identify raters as construct-irrelevant error [23–25], our results represent validity evidence supporting the internal structure for a rater-based written test in OMPT certification. Although the effect of rater training on their rating performance was not the focus of the present study, one possible explanation for the observed rater consistency may be raters’ familiarity with the CHAT gained through a standardized training module. To strengthen previous research on the effect of training efforts for raters of clinical examinations [25], future work should further examine the effectiveness of rater training on rating performance for short-answer tests.

Importantly, the cognitive workload associated with the use of the CHAT differs from the rating task involved in performance-based assessments such as an OSCE, which may further explain the low rater variance found in the present study. In the case of the CHAT, while the rater is required to select, detect and process relevant aspects of candidates’ written responses that pertain to clinical reasoning, these tasks are not time-limited. Rather, rating tasks for written tests afford raters more than a single opportunity to review candidates’ written responses prior to categorizing them; thus, the rating tasks associated with the CHAT are not only feasible and acceptable to examiners [12], it is arguably less dependent on raters’ working memory and thus is less prone to rater idiosyncrasies resulting from the use of one’s memory. Moreover, because short-answer tests are not susceptible to the same measurement errors associated with impression formation in other rater-based assessments [26], raters’ accuracy in categorizing candidates’ written responses is not compromised in the same manner as in an OSCE.

Notably, the generalizability coefficients for each of the individual rater pairs were lower than that for all raters combined; however, these reliability estimates were still within an acceptable range [17]. It is possible that varying levels of prior experience with the CHAT gained through raters’ involvement in the preceding feasibility study, may have contributed to the observed variability in rater performance. Moreover, raters within each rater pair may have applied different standards of stringency or leniency for the same candidate, thus contributing to the observed variance components attributed to raters within the rater pairs [19, 27]. The use of Rasch modeling in future research could provide important data regarding the stringency or leniency of raters in larger samples of candidates, as would inviting examiners to assess the same short-answer tests on different occasions (test-retest reliability). Data from such studies would potentially strengthen the reliability of scores generated from the CHAT.

Decision studies (D studies) revealed that increasing the number of raters would not impact significantly on the overall reliability of test scores generated from the CHAT. Similar to other high-stakes assessment contexts [28], increasing the number of raters in the present study resulted in minimal impact on overall reliability as compared to increasing the number of test questions, suggesting an issue of context specificity. The assessment of clinical reasoning is also context specific and demand adequate sampling in order to provide an appropriate assessment of this construct [29]; thus, while results from this study offer evidence of validity to support the use of scores derived from the CHAT, other assessment formats are needed to triangulate data concerning candidates’ clinical reasoning competence. Although it is not feasible to sample from multiple occasions for the same short-answer test for each candidate, our findings affirm the need to collect evidence of clinical reasoning competence through other means such as observations of candidate performance during supervised practice in the clinical setting, as well as the oral practical and multiple-choice examinations. Future studies aimed at examining the relationship between scores from the CHAT and scores from other assessment procedures that measure clinical reasoning are warranted [5].

While the variance attributed to *question* was minimal in the omnibus analysis, the corresponding variance differed across the novice and PT groups*.* This reflects the different types of questions contained within the short-answer test as well as the varying levels of question difficulty. Upon further analysis of internal consistency, it appears that 4 test questions may contain assessment items that are not measuring the same underlying construct (clinical reasoning) to an acceptable degree (Table 5). Specifically, redundancies exist amongst items assigned to several test questions related to hypothesis generation and management strategies. Recommendations to focus on assessment of candidates’ knowledge organization and their ability to integrate new clinical information may serve to guide further item analysis to enhance the validity of the internal structure of the CHAT [30]. At the same time, it is important to note that a careful balance must be struck between improving variance and reliability of examination scores and maintaining a reasonable standard and relevancy of the exam content.

**Table 5:** Internal consistency analysis

	# of Items	Mean (SD)	Cronbach’s Alpha	Corrected Item-Total Correlation	Cronbach’s Alpha if item deleted
Questions with Cronbach’s alpha <0.70					
Q2 (Section one). The table below describes different mechanisms that may be influencing the patient’s pain. Based on the information provided in the subjective examination, list the evidence, if any that would be most indicative of each pain mechanism. Consider all areas of pain (8 marks)	3	5.66/8 (1.39)	0.667	0.433–0.513	0.536–0.631
Q4 (Section one). Which category best describes the overall irritability of this patient’s condition (Mild, Mild-moderate, Moderate, Moderate-Severe, Severe). Justify your answer with 4 pieces of evidence from the subjective examination. What are the implications of this for the physical examination? (3 marks)	3	1.75/3 (0.58)	0.616	0.206–0.781	0.116–0.799
Q6 (Section one). List 3 subjective examination findings that would indicate caution must be observed during the objective examination. Explain why. (3 marks)	2	1.60/3 (0.71)	0.683	0.528	n/a
Q2 (Section two). List 2 favourable and 2 unfavourable prognostic indicators for this patient and considering these, describe your predictive outcome. (3 marks)	2	2.0/3 (0.56)	0.497	0.34	n/a
Questions with item-total correlations > 0.70					
Q7 (Section one). After reading the subjective data, list the 2 (most likely) clinical hypotheses and provide 3 subjective findings to support each hypothesis. (3 marks)	2	1.69/3 (0.50)	0.843	0.733	n/a
Q8 (Section one). Based on the subjective examination you have developed two clinical hypotheses. Provide 4 key elements of your physical examination and under each element state 2 of the most relevant tests you would perform and explain how these would help you confirm or negate your hypotheses. (8 marks)	2	5.01/8 (1.59)	0.857	0.753	n/a
Q9 (Section one). What are 2 outcome measurement tools or screening tools that you would use to monitor this patient’s progress and provide your rationale for choosing them. (2 marks)	2	1.57/2 (0.46)	0.858	0.76	n/a
Q1 (Section two). Provide your main hypothesis for this patient’s clinical picture. Outline in detail your rationale and justification for this hypothesis with consideration of the evidence from both the subjective and objective examination. (8 marks)	2	5.57/8 (1.57)	0.882	0.789	n/a
Q4 (Section two). Indicate your primary functional goal as it relates to the activity limitations and participation restrictions and select 2 problems that would be the most relevant to address. Include your treatment goal for each problem and the testing criteria you would use to monitor change. (6 marks)	5	3.76/6 (1.44)	0.931	0.700–0.903	0.89–0.935
Q5 (Section two). Outline in detail the management strategies you would use over the first two treatments under the following headings: manual therapy, exercise, education and other. Include your rationale. (8 marks)	7	5.11/8 (1.72)	0.935	0.730–0.879	0.917–0.935
Q6 (Section two). Outline in detail your progression of subsequent treatments to discharge, addressing all the identified problems and provide your rationale. Use the following headings: manual therapy, exercise, education and other. (8 marks)	6	4.84/8 (1.83)	0.931	0.709–0.917	0.903–0.938

Finally, the large variance associated with sources of error not incorporated in our G studies raise concerns regarding other error sources that may affect measurement precision. Given the diverse range of clinical reasoning strategies employed by physical therapists [20], it is possible that rater idiosyncracies and their own cognitive limitations may hinder the acquisition and processing of a wide variation in test responses resulting in undesired rater idiosyncrasies. Thus, the role that cognitive capacity plays during the rating task should be the target of future investigations. Since rater cognition during the information acquisition and processing phases have been hypothesized to be relevant constraints in other rater-based assessments [31], efforts aimed at understanding the reasons for variations in raters’ selection and detection will likely further improve measurement precision.

### Study limitations

Because we aimed to generalize assessment results generated by raters that are representative of all OMPT raters in Canada, we recruited raters with varying levels of examination experience from the existing pool of Canadian examiners. Owing to issues of feasibility, we were unable to conduct a fully crossed design (p x r) whereby all candidates’ test papers were rated by all participating raters. Given the current resource constraints, only two raters were employed to assess each test paper. Although this limits how closely we can model the structure of the data in generalizability studies, the facets that have been collapsed (for example, *rater*) were not targets of our investigation. Moreover, our study investigated test scores from a single examination administration and thus may result in somewhat inflated generalizability coefficients; however, this study establishes preliminary data to guide future validity studies to further examine the potential contributors to measurement error.

## Conclusion

Our study results provide validity evidence supporting the internal structure of the CHAT and highlight its suitability for practicing physiotherapists pursuing post-graduate OMPT certification. Important considerations for examining measurement errors associated with rater-based short-answer tests were also emphasized. Future studies should attend to two additional validity constructs not previously examined, namely the relationship of CHAT scores to other variables and consequences of decisions based on scores derived from the CHAT. Results from the present study provide important information about the nature and extent of the sources of error associated with the CHAT, as well as practice and research implications for written tests of clinical reasoning.

## Abbreviations

CHAT, case history assessment tool; D study, decision study; G study, generalizability study; G theory, generalizability theory; MFRM, multi-faceted rasch modelling; OMPT, orthopaedic manual physical therapy; OSCE, Objective Structured Clinical Examination; PT, physical therapist
